# Three-Dimensional Genome Organization and Function in *Drosophila*

**DOI:** 10.1534/genetics.115.185132

**Published:** 2016-12-28

**Authors:** Yuri B. Schwartz, Giacomo Cavalli

**Affiliations:** * Department of Molecular Biology, Umeå University, 901 87 Umeå, Sweden; † Human Genetics, Centre National de la Recherche Scientifique, UPR1142 and University of Montpellier, 34396 Montpellier Cedex 5, France

**Keywords:** FlyBook, genome architecture, chromatin insulators, epigenetics

## Abstract

Understanding how the metazoan genome is used during development and cell differentiation is one of the major challenges in the postgenomic era. Early studies in *Drosophila* suggested that three-dimensional (3D) chromosome organization plays important regulatory roles in this process and recent technological advances started to reveal connections at the molecular level. Here we will consider general features of the architectural organization of the *Drosophila* genome, providing historical perspective and insights from recent work. We will compare the linear and spatial segmentation of the fly genome and focus on the two key regulators of genome architecture: insulator components and Polycomb group proteins. With its unique set of genetic tools and a compact, well annotated genome, *Drosophila* is poised to remain a model system of choice for rapid progress in understanding principles of genome organization and to serve as a proving ground for development of 3D genome-engineering techniques.

THE first metazoan whole genome sequence was completed in *Drosophila* *melanogaster* only 16 years ago (Adams *et al.* 2000). The 180-Mb fly genome, is packaged into sex chromosomes, two large metacentric autosomes, and a smaller heterochromatic autosome (chromosome 4). Each of the large chromosomes has a DNA molecule of ∼5 cm, but it has to fit into a nucleus of an average diameter of ∼5 μm. Therefore, chromosomes must be condensed thousands of times on the linear scale to fit into the nucleus. Importantly, chromatin compaction must be achieved in a way that allows access to the machineries that carry out DNA-dependent processes, such as transcription, replication, recombination, and repair. This is achieved thanks to chromatin folding into a hierarchy of structures, such as nucleosomes, nucleosome fibers, chromosome domains, and chromosome territories (Cavalli and Misteli 2013). Recent data have suggested that this organization is an important contributor to the regulation of gene expression. In particular, epigenomic maps of histone modifications and chromatin factors have shown that the genome is partitioned into domains that have a limited diversity in their chromatin composition (Filion *et al.* 2010; Kharchenko *et al.* 2011; Ho *et al.* 2014). Furthermore, analysis of genome-wide chromosome contact data by Hi-C technology showed that epigenomic domains correspond to physical domains of chromosome folding (Hou *et al.* 2012; Sexton *et al.* 2012). These physical domains have also been identified in mammals and dubbed as topologically associating domains (TADs) (Dixon *et al.* 2012; Nora *et al.* 2012). They are also present in other animal species and, to some extent, they can also be found in yeast and plants (Grob *et al.* 2014; Hsieh *et al.* 2015), suggesting that they represent a conserved mode of chromosome organization (Ciabrelli and Cavalli 2015; Sexton and Cavalli 2015).

Research in *Drosophila* has greatly contributed to understanding the importance of three-dimensional (3D) genome organization for its function. Genetic evidence for long-range effects in the regulation of gene expression was linked to a role of heterochromatin in gene silencing (Cohen 1962). The discovery of the transvection phenomenon by Ed Lewis revealed that interchromosomal interactions may modulate gene expression (Lewis 1954). These interactions were later shown to mediate not only transcriptional activation but also repression and to be mediated either by heterochromatin (Csink and Henikoff 1996; Dernburg *et al.* 1996) or Polycomb components (Pirrotta and Rastelli 1994; Zink and Paro 1995; Bantignies *et al.* 2003). Another class of chromatin components that affect gene expression in *cis*, and in *trans*, were dubbed as chromatin boundaries or insulators: regions of several hundred base pairs that are bound by a variety of components (Holdridge and Dorsett 1991; Kellum and Schedl 1991; Geyer and Corces 1992). Many of these findings were later shown to apply to other species of animals and plants, even though their detailed molecular mechanisms differ to some extent. Below, we will describe general features of the architectural organization of the fly genome, providing historical background and insights from recent studies. We will then describe two main regulators of genome architecture, namely insulator components and Polycomb group proteins. Finally, we will outline relevant open questions and provide perspectives into future directions that remain to be explored.

## Early Evidence for a Role of Chromosome Architecture in Fly Genome Function

Although recent technologies suggest that 3D chromosome organization may have regulatory roles, *Drosophila* genetics had indicated that this may be the case for many decades. First hints toward this came with the description of the phenomenon of position-effect variegation (Muller 1930). Initially described for the *white* gene, this phenomenon was later shown to extend to many other genes and to consist of a clonal gene silencing effect, which was found to depend on the proximity of the silenced gene to heterochromatin (Lewis 1945, 1950; Spofford 1959, 1967; Cohen 1962). Heterochromatin was first discovered in microscopy preparations by Emil Heitz in 1928, who defined it as a genetically inert part of the genome, which remains heavily condensed throughout the cell cycle (Heitz 1928). A plethora of later studies showed that heterochromatin is formed by large genomic domains rich in repetitive elements and is transcriptionally silent (Dejardin 2015). The variegated eye phenotype was of seminal importance in the chromatin field, since it allowed the development of genetic screens for modifiers of position-effect variegation (Reuter and Wolff 1981). These screens led to the identification of critical components of heterochromatin, such as Su(var)3-9 (Tschiersch *et al.* 1994), and provided a genetic basis for the regulatory function of post-translational histone modifications. These early findings showing that cytological proximity to heterochromatin induced variable degrees of gene silencing were later extended by many other works, making a strong case for long-range chromosomal effects in the regulation of gene expression (Lewis 1945, 1950; Cohen 1962). Heterochromatin formation was proposed to involve a large number of proteins, forming macromolecular complexes whose action would follow a mass-action law (Tartof *et al.* 1989). The relative concentration of the various components of heterochromatin would determine the extent to which it would silence the genes immediately adjacent to the pericentromeric regions, and the cell-to-cell variability in these components might explain the variable extent of silencing observed in position-effect variegation.

Position effects are not limited to heterochromatin, however. The wide use of *P*-element-mediated transformation (Rubin and Spradling 1982), which results in semirandom integration of reporter constructs in the *Drosophila* genome, was instrumental in studying these effects. Used for over two decades until the advent of site-specific integration techniques (Bischof *et al.* 2007), it effectively sampled position effects at hundreds of thousands of genomic locations. A common observation from transgenic reporters carrying the *white* gene, is that the eye color varies considerably in different lines. This depends on the effect exerted by regulatory elements located at the site of transgene insertion. This phenomenon of position effect suggested not only the idea that genes may be subjected to the influence of their flanking chromatin, but also that, in the genome, specific mechanisms must exist to normally protect gene regulation from illegitimate effects of surrounding chromatin.

In addition to relatively short-range effects that involve genes and regulatory regions from the same genomic neighborhood, higher-order chromatin structures can have long-range effects on distant locations in the same or even different chromosomes. In *Drosophila*, a frequent case of long-range chromatin contacts that can result in gene regulation depends on the property of somatic homologous chromosome pairing. That homologous chromosomes can pair was suggested by microscopy study from the beginning of the 20th century, but genetic studies clearly substantiated the regulatory nature of this phenomenon in the 1950s. Ed Lewis coined the term “transvection” in 1954 to indicate situations in which the phenotype of a given genotype can be altered solely by disruption of somatic (or meiotic) pairing. Originally, Ed Lewis identified transvection at the bithorax complex (Lewis 1954). Independently, Madeleine Gans had identified another case of this phenomenon 1 year earlier, while studying the *zeste* locus and its regulatory effects on the *white* gene (Gans 1953). Later, many other cases of transvection were identified at other loci, including *decapentaplegic*, *eyes absent*, *vestigial*, and *yellow*, and representing cases of gene activation as well as repression (Pirrotta and Rastelli 1994; Wu and Morris 1999; Duncan 2002). In the case of activation, the typical case of transvection is when enhancers located on a chromosome carrying a mutation in their target promoter can activate the promoter of the same gene on the homologous chromosome (Morris *et al.* 1999). In the case of silencing, the term pairing-sensitive silencing (PSS) is often used instead of transvection (Kassis *et al.* 1991). Pairing effects have been documented in the case of Polycomb-mediated gene silencing and heterochromatin. Polycomb proteins were originally identified as repressors of homeotic genes (Lewis 1978), although later they were shown to repress a large number of genes, many of which are involved in developmental patterning and in the regulation of cell proliferation (Grimaud *et al.* 2006b; Schwartz and Pirrotta 2007; Schuettengruber and Cavalli 2009). They are targeted to chromatin at specific regions called Polycomb response elements (PREs) (Entrevan *et al.* 2016). When these PREs are inserted in transgenes flanking a reporter such as the mini-*white* gene, they silence it in a variegated manner. Silencing is often enhanced when the transgene is in a homozygous state, compared to the heterozygous condition (Pirrotta and Rastelli 1994; Zink and Paro 1995). In some cases, Polycomb-regulated transgenes inserted at different genomic locations also associate. This leads to stronger silencing and shows that *trans*-interactions are not restricted to homologous sites (Pal-Bhadra *et al.* 1997; Muller *et al.* 1999; Bantignies *et al.* 2003). Another silencing system linked to chromosomal *trans*-interactions is heterochromatin. In *Drosophila*, similar to other organisms, the telomeric and centromeric regions of each chromosome are flanked by large blocks of repetitive sequences that assemble into heterochromatin. In particular, pericentromeric heterochromatin blocks can span over 10 Mb of DNA. These blocks establish *trans*-interactions, such that they form a cytologically visible structure called the chromocenter (Hiraoka *et al.* 1993). One particular case of heterochromatin-mediated gene silencing is the *brown^Dominant^* (*bw^D^*) allele, in which a block of ∼2 Mb of heterochromatin containing the AAGAG satellite sequence is inserted in the coding region of the *bw* gene. Strikingly, when the *bw^D^* allele is heterozygous to a wild-type (WT) copy of *bw*, this copy is repressed by *bw^D^*. The repression involves a contact between the two alleles in *trans*, and the repositioning of the WT allele from its normal nuclear location toward centromeric heterochromatin (Csink and Henikoff 1996, 1998; Dernburg *et al.* 1996). Finally, in addition to transcriptional repressors or activators, insulator proteins also establish long-range contacts (Gerasimova *et al.* 2000). In this case, the contacts seem to orchestrate genome architecture and, rather than directly inducing or repressing the specific contact loci, they seem to modulate gene expression by optimizing the spatial organization of the genome (Gomez-Diaz and Corces 2014).

From the early evidence described above, it became clear that chromatin and nuclear architecture must play an important role in regulating all aspects of genome function. Nevertheless, the field has progressed relatively slowly for decades, due to the paucity and the technical challenges of the methods to study the 3D architecture. The very first interesting observations came from the study of polytene chromosomes of the salivary gland cells. Polytene chromosomes have always been an invaluable asset for *Drosophila* research. Initial studies using first light, and then electron, microscopy allowed to partition the *Drosophila melanogaster* genome in 102 main cytological divisions, further divided into six subsections each, and even further in variable numbers of subdivisions. Systematic *in situ* hybridization of genomic libraries to polytene chromosomes allowed assignment of each gene to a cytological localization (Kafatos *et al.* 1991; Hartl *et al.* 1992). The development of protein immunostaining and simultaneous application of *in situ* hybridization enabled localization of a protein of interest to specific gene loci, the approach that inspired contemporary chromatin profiling studies (Zink and Paro 1989; Clark *et al.* 1991; Stephens *et al.* 2004; Dejardin *et al.* 2005). Electron and confocal microscopy was also applied to salivary gland nuclei, allowing the reconstruction of the architecture of polytene chromosomes (Agard and Sedat 1983; Semeshin *et al.* 1985a,b, 1989). Although the information gained from these studies may not be easy to generalize because of the polyploid nature of salivary gland nuclei, this work stimulated the development of sophisticated microscopy tools to study diploid cells. The use of fixed tissue as well as *in vivo* techniques tracking GFP-tagged chromatin components and individual genes identified many general principles of *Drosophila* chromatin organization and dynamics (Marshall *et al.* 1997; Gerasimova *et al.* 2000; Harmon and Sedat 2005; Cheutin and Cavalli 2012).

As we discuss in detail below, the pace of our progress toward understanding the 3D architecture of the *Drosophila* genome was greatly boosted by the advent of genomic techniques. Those allowed systematic mapping of multiple chromatin components and histone modifications (Schwartz *et al.* 2006, 2012; Filion *et al.* 2010; Negre *et al.* 2010a; Kharchenko *et al.* 2011) and led to the development of methods to map 3D chromatin contacts in live cells and with high precision (Hou *et al.* 2012; Sexton *et al.* 2012).

## Partitioning of the *Drosophila* Genome into Domains with Discrete Chromatin Types

The striking banding pattern of *Drosophila* polytene chromosomes visually demonstrates that interphase chromosomes are partitioned into stable chromatin domains (Zhimulev 1996). However, which chromatin features underlie the pattern? Could unique combinations of post-translationally modified histones or specific sets of nonhistone proteins define chromatin domains? First attempts to map components of the Polycomb repressive system by chromatin immunoprecipitation (ChIP) coupled with hybridization of ChIP products to high-resolution genomic tiling microarray suggested that this hypothesis is correct, at least to some extent (Negre *et al.* 2006; Schwartz *et al.* 2006; Tolhuis *et al.* 2006). Thus, genes repressed by Polycomb mechanisms reside within broad domains enriched with histone H3 trimethylated at lysine 27 (H3K27me3). Embedded within H3K27me3 domains are one or several PREs, which appear as narrow high-affinity binding platforms for Polycomb proteins (Schwartz *et al.* 2006). Although instructive, Polycomb-controlled chromatin domains cover only a small part of the genome. What about the rest? In the pioneering attempt to address this question, Filion *et al.* (2010) used DNA adenine methyltransferase identification (DamID) technology to map genome-wide distributions of 53 *Drosophila* nonhistone chromatin proteins representing some of the histone-modifying enzymes, proteins that bind specific histone modifications, general transcription machinery components, nucleosome remodelers, structural components of chromatin, and a set of sequence-specific transcription factors. In DamID, the bacterial Dam is fused to a chromatin protein of interest and leaves a stable adenine-methylation mark at the *in vivo* interaction sites of the chromatin protein (van Steensel and Henikoff 2000). DamID has lower resolution compared to ChIP, but does not require large numbers of high-quality antibodies. Using principal component analysis (Jolliffe and Cadima 2016) of binding profiles of 53 chromatin proteins followed by hidden Markov model fitting (Schuster-Bockler and Bateman 2007), Filion *et al.* (2010) were able to partition the *Drosophila* genome into domains of five principle chromatin types, which they color coded as blue, green, black, red, and yellow. In this classification, the blue chromatin corresponds to loci regulated by Polycomb proteins and the green chromatin corresponds to pericentromeric regions enriched in HP1 and Su(var)3-9. Even at such coarse-grained partitioning, the chromatin of transcriptionally active genes is represented by two distinct (red and yellow) states, suggesting that gene expression is accompanied by multiple distinct chromatin remodeling processes. Finally, in this classification, the major part of the transcriptionally inactive genome was assigned to black chromatin, with poorly understood and possibly repressive properties.

Shortly after, followed a comprehensive analysis of the fly chromatin landscape by the large-scale model organism encyclopedia of DNA elements (modENCODE) project. This project produced detailed ChIP profiles of chromatin components and mapped *Drosophila* transcripts and small RNAs (modENCODE Consortium *et al.* 2010). With this information and a machine-learning approach similar to that of Filion *et al.* (2010), the genome of interphase *Drosophila* cells was partitioned into nine chromatin types, characterized by unique combinatorial patterns of 18 histone modifications (Kharchenko *et al.* 2011). In agreement with the “five-color” chromatin partitioning, more distinct chromatin types were associated with transcriptionally active genes. Thus, active transcription start sites (TSSs), exons, and introns of transcribed genes were each associated with distinct chromatin types. In addition, active genes on the X chromosome of male cells were associated with a specific chromatin state rich in histone H4 acetylated at lysine 16 (H4K16ac). The latter reflects the process of dosage compensation where expression of genes on the single male X chromosome is upregulated roughly twofold (Lucchesi and Kuroda 2015). The nine-state model also distinguishes the two kinds of heterochromatin-like types of chromatin, which differ in the extent of di- and trimethylation of lysine 9 of histone H3 (H3K9me2/me3). Similar to the five-color chromatin partitioning, a large fraction of the transcriptionally inactive genome is assigned to a “void” chromatin type low in any of the measured histone modifications. More complex models that use probability of the presence or absence of individual histone modifications can partition the genome into even larger sets of chromatin types. For example, using the same data on combinatorial patterns of 18 histone modifications, the chromatin was partitioned into 30 different types (Kharchenko *et al.* 2011). Compared to nine-type partitioning, such a fine division does not necessarily bring many new biological insights but can, for example, identify distinct chromatin signature of transcriptional elongation in genes embedded with pericentric heterochromatin (Kharchenko *et al.* 2011; Riddle *et al.* 2011). We should note that, regardless of the complexity, any general partitioning of the genome remains an approximation. For example, the exact positions of the “boundaries” between distinct chromatin states depend on the parameters of computational algorithms and some fine chromatin features (*i.e.*, composition of individual nucleosomes within a more homogeneous neighborhood) may get averaged out during the analysis. Therefore, although any two genes or regulatory elements assigned to the same chromatin type are likely to share many properties, their functional behavior may still be different.

To conclude, the distribution of post-translationally modified histones and nonhistone chromatin components defines distinct combinatorial patterns that partition the genome into domains with distinct chromatin types. At a chromosome scale view, we can see pericentric regions (sometimes called heterochromatin) and chromosome 4 embedded within chromatin domains rich in H3K9me2/me3 and HP1 and the rest of the genome (sometimes collectively referred to as euchromatin) represented by ∼10- to 200-kb domains of black/void chromatin alternating with similar-sized domains enriched in H3K27me3/Polycomb proteins or clusters of short domains with chromatin types characteristic of active genes. As we discuss in the following section, the segmentation of the linear *Drosophila* genome into distinct chromatin types is in many ways connected to its architectural organization in 3D space.

## The Hierarchical Nature of Fly Genome Architectural Organization

The utilization of Hi-C technology (Lieberman-Aiden *et al.* 2009) to map in an unbiased manner genome-wide chromatin contacts has allowed for the first time to deduce basic underlying principles of genome folding in different species. In its original version, this method, applied at a shallow sequencing depth, allowed the identification of two main compartments, an active or A type, including large multimegabase-sized regions that are dense in active genes, and an inactive or B type, which includes similar sized regions with low levels of gene expression. When a variation of this method was applied to the fly genome and sequencing power was increased massively, in addition to active and inactive compartments, smaller domains with a size on the order of 100 kb on average were readily detected (Sexton *et al.* 2012). The distinguishing feature of these domains is that high levels of interaction are found among all fragments within each domain, whereas interdomain interactions have lower frequency, and sharp boundaries define the points at which interaction frequencies change. These regions were therefore called physical domains. Increasing the sequencing depth allowed detection of similar regions, TADs, in mammalian genomes (Dixon *et al.* 2012; Nora *et al.* 2012). In contrast to *Drosophila*, the size of TADs in human and mice is on the order of 1 Mb on average. In both human and flies, inactive TADs roughly correspond to regions of strong attachment to the nuclear lamina [lamina-associated domains (LADs)], whereas active TADs are characterized by lower frequencies of lamina association (Pickersgill *et al.* 2006; Guelen *et al.* 2008; Peric-Hupkes *et al.* 2010; Dixon *et al.* 2012). Furthermore, TADs correlate even better with domains of a defined timing of DNA replication during the S phase of the cell cycle, with active TADs equivalent to early replicating domains and heterochromatic TADs equivalent to late replicating domains (Ryba *et al.* 2010; Pope *et al.* 2014). This suggests that the architectural partitions of the genome correspond to their physical and functional organization. Recently, different variants of the Hi-C method have been applied in different species, both in the eukaryote and the prokaryote domains. Each of these variants has advantages and limitations, which should be carefully considered when designing experiments and interpreting their results, as reviewed and discussed elsewhere (Sati and Cavalli 2016). However, the existing work shows that Hi-C is a powerful and robust method, which enables reliably detecting chromatin interactions even when present in only a few percent of the cells in the sample, as in the case of very long-distance interactions in the same (Sexton *et al.* 2012) or in different chromosomes (Schoenfelder *et al.* 2015). In all cases, chromosomes do not fold as generic polymer structures but instead they possess some kind of specific domain organization (Sexton and Cavalli 2015). Nevertheless, nematode and plant Hi-C data show that, although some domain structure exists, strongly demarcated TADs are lacking (Sexton and Cavalli 2015). In yeast, small physical domains exist of <10-kb average size, whereas in some bacteria, large domains in the megabase size range have been identified. A general rule for eukaryotic genomes seems to be that, when physical domains exist, they seem to scale with the average size of genes and of the genome. Species with larger genes and genomes tend to have larger individual TADs. Bacteria do not seem to follow this rule and, possibly, TADs are not only linked to gene function but also to other functions like genome replication and segregation (Badrinarayanan *et al.* 2015; Marbouty *et al.* 2015; Le and Laub 2016).

**Figure 1:**
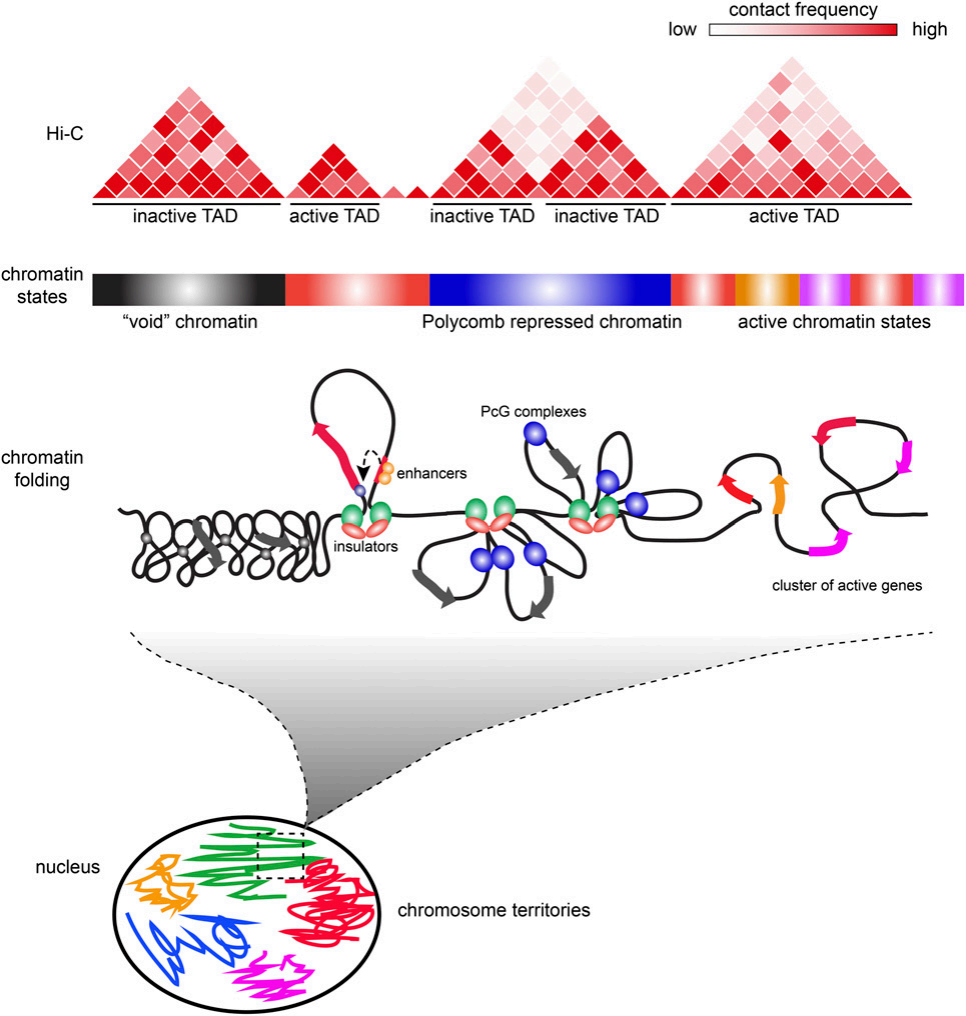
Hierarchies of fly genome architecture. Chromosomes are extensively folded to fit inside the cell nucleus. Each chromosome occupies its own volume (chromosome territory, shown schematically in different colors); however, these volumes partially intersect allowing for interchromosomal interactions. At finer scale, chromatin fibers are partitioned into domains with different degrees of folding and different regimes of chromatin contacts within domains (TADs). The partitioning into domains is in part defined by the differences in the composition and properties of underlying chromatin as well as transcriptional activity. For example, void and Polycomb (PcG)-repressed chromatin has more internal contacts than chromatin of active genes. Insulator elements demarcate some of the TADs by forming loops that inhibit chromatin contacts across domain boundaries. Topological domains correlate with segmentation of the linear *Drosophila* genome into chromatin types with distinct histone modifications. The contact matrix of a virtual Hi-C experiment illustrates how partitioning into TADs is assayed.

One of the main observations from mammalian Hi-C studies is that the majority of TADs are invariant in different cell types and also strongly conserved in evolution (Dixon *et al.* 2012). Comparison of Hi-C profiles between fly embryos and Kc cells revealed a similar robustness of fly TADs among cell types (Hou *et al.* 2012) and even between diploid and polytene tissue (Eagen *et al.* 2015), suggesting that these domains represent a chromosome organizational blueprint of most fly cells. But what defines these domains and what are the forces responsible for their formation? A striking observation from the original Hi-C study is that there is a strong correspondence between TADs and epigenomic marks (Figure 1). Typically, each TAD has a dominant type or combination of epigenetic marks, corresponding to a specific functional demarcation. Inspection of this correspondence revealed four different types of TADs, including one active and three different inactive classes.

Active TADs include many transcriptionally active genes and their regulatory regions. Therefore, they correlate with open chromatin marks, such as acetylated histones, as well as histone marks typical of enhancers, promoters, but also coding regions (Ho *et al.* 2014). Compared to other types, active TADs have a distinctive feature that can be measured by quantifying the frequency of contacts relative to the distance of any anchor point within a TAD. A universal feature of chromosomes and, actually, of any polymer, is that each of its monomers contacts more frequently other monomers that are close on the linear scale, compared to those that are located far away (Jost *et al.* 2014). For active TADs, the contact frequency decay as a function of linear distance is faster than for other types of TADs. This could indicate that inactive chromatin is more condensed than the active counterpart. Recent superresolution microscopy studies have indicated that this might indeed be the case (Boettiger *et al.* 2016). However, another contribution to this observation may come from the dynamics of chromatin motion. Indeed, chromatin moves with a specific speed inside the nucleus, which depends on chromatin type and position within the chromosome (Heun *et al.* 2001; Cheutin and Cavalli 2012). It might thus be possible that, on average, active chromatin has faster dynamics and that chromatin contacts are shorter lived than in other types of chromatin. Of note, the agent used for capturing contacts, formaldehyde, has slow kinetics (tens of minutes of cross-linking are required in Hi-C protocols) compared to the kinetics of motion and the average residence time of many proteins on chromatin (Misteli 2001; Cheutin *et al.* 2003). Therefore cross-linking might be less efficient in this type of chromatin compared to more inactive chromatin types in which the average duration of contacts might be longer. More work is required to investigate this point (Gavrilov *et al.* 2015).

In addition to active TADs, three types of inactive TADs have been identified. The first corresponds to Polycomb repressed loci enriched in histone H3 trimethylated at lysine 27 (H3K27me3) (Hou *et al.* 2012; Sexton *et al.* 2012). Polycomb TADs represent ∼10% of the fly genome and contain a large number of developmental genes, many of which encode transcription factors involved in patterning. These physical domains have a counterpart in microscopy, as antibody staining and GFP fusion protein detection had previously identified a discrete number of staining signals, also called PcG foci (Cheutin and Cavalli 2014). Many of these foci correspond to spatial clustering of binding sites within an individual domain (Lanzuolo *et al.* 2007) or to long-range interactions among different PcG domains (Grimaud *et al.* 2006a; Bantignies *et al.* 2011). A second type of silent TADs contains heterochromatin. This is mainly located at pericentromeric regions as well as telomeric regions of the chromosomes. In Hi-C, distinctive interchromosomal contacts among pericentromeric heterochromatin are detected. These contacts can be seen even when strictly unique genome sequences are analyzed and thus they do not represent artifacts due to the highly repetitive nature of pericentromeric sequences. Moreover, subtelomeric regions also contact each other and independently of pericentromeric regions (Sexton *et al.* 2012). A few other euchromatic regions carrying the same histone modifications as heterochromatin, namely H3K9me2 and H3K9me3, also build inactive domains of the same kind; however, they are limited to a relatively small set of regions. One critical issue to keep in mind when considering this type of chromatin is that all epigenomic maps until now have been inevitably restricted to the unique portion of the genome, since repeated parts of the genome cannot be physically mapped to a specific locus. This means that the one-third of the fly genome containing repeats is invisible to Hi-C. It would of course be important to analyze chromatin composition and architecture of this portion as well, since it is likely to influence genome function in a major way. As discussed above, genes in the vicinity of large heterochromatic blocks, either on the linear scale or spatially, can be repressed by heterochromatic components. Since hundreds of full-length or defective transposons are inserted in the fly genome, it is possible that many of them might regulate genes that either reside in the vicinity or are associated in the 3D space of the nucleus. Indeed, the possibility of 3D organization for repetitive regions beyond pericentric or telomeric repeats is supported by careful analysis of embryonic Hi-C data. This analysis indicates that gene clusters encoding Piwi-interacting small RNAs form preferential contacts (Grob *et al.* 2014). It will be interesting to analyze whether these contacts have a regulatory value. A final type of repressive chromatin domain is defined as void or black chromatin. This encompasses a large portion (up to 50% of euchromatin) of the genome, characterized by low or no transcriptional activity and low or absent histone modifications of any kind (assuming that the full catalog of modifications is known) (Filion *et al.* 2010; Sexton *et al.* 2012; Ho *et al.* 2014). The initial mapping of chromatin factors to this genomic portion did, however, identify low levels of various chromatin components that are shared with Polycomb and, to a lesser extent, heterochromatin domains (Filion *et al.* 2010; Ho *et al.* 2014). This suggests the possibility that black chromatin may represent a passive inactive state which, upon selective recruitment of specific components depending on developmental cues, can switch into a Polycomb, a heterochromatic, or an active state. On the other hand, it is also likely that, when mapping in different cell types or developmental stages, genomic regions shift from black to active to accommodate changes in gene expression. Evidence for these kinds of changes is, however, sparse and it will be important to address these issues in the future.

One important feature of genome folding is that, in addition to domains, a yet higher-order level of chromatin folding involves interactions among TADs. In embryos, a clear tendency for TADs of the same kind to interact preferentially was detected (Sexton *et al.* 2012), suggesting that direct protein–protein interactions among components decorating each of the types may be causally linked to these long-distance interactions. Lower but discernible interactions also exist between the three types of chromatin domains, forming a repressive compartment in the fly genome, whereas active and any of the repressed domains segregate in clearly different nuclear compartments (Sexton *et al.* 2012). This tendency is even exaggerated in mammalian genomes, where several adjacent TADs often behave like a single multimegabase-sized domain when considering very-long-range interactions (Lieberman-Aiden *et al.* 2009; Rao *et al.* 2014). These interactions involving large domains are probably at the base of the formation of the chromosome territories, which are detected by fluorescent *in situ* DNA hybridization (DNA FISH) using whole chromosome probes. In such DNA FISH experiments, each chromosome appears to occupy a distinct portion of the nuclear space. In addition to these generic interactions, other much more specific interactions may occur between individual regulatory regions in the genome. First, a recent survey of interactions between a large set of embryonic enhancers and promoters identified specific interactions not only between each enhancer and its cognate neighboring gene promoter target, but also include very-long-range interactions with genes located hundreds of kilobases and several TADs away. Furthermore, these interactions are largely preset, before the time at which the target gene of each of these enhancers will start to be activated (Ghavi-Helm *et al.* 2014). Similar observations have been made when studying mammalian embryonic stem (ES) cell differentiation, suggesting that architectural organization may be a critical requirement to set up regulatory landscapes at least for a subset of genes. Second, as discussed above, regulatory processes such as transvection can involve interactions not only in *cis*, but also in *trans*, among different chromosomes. In some cases, these interactions have also been detected in microscopy (Ronshaugen and Levine 2004), but how widespread they are in the genome is not clear. Third, a specific type of long-range contacts may involve a subset of the sites that specify the borders of TADs. Thus, preferential interactions have been reported for subsets of TAD borders that contain binding sites for insulator factors in flies (Hou *et al.* 2012; Van Bortle *et al.* 2014), as well as for CTCF sites in humans (Rao *et al.* 2014). Understanding the role of these long-range contacts for regulation of specific genes is an important task for future research.

## Defining the Borders of Topological and Functional Chromosomal Domains

**Figure 2:**
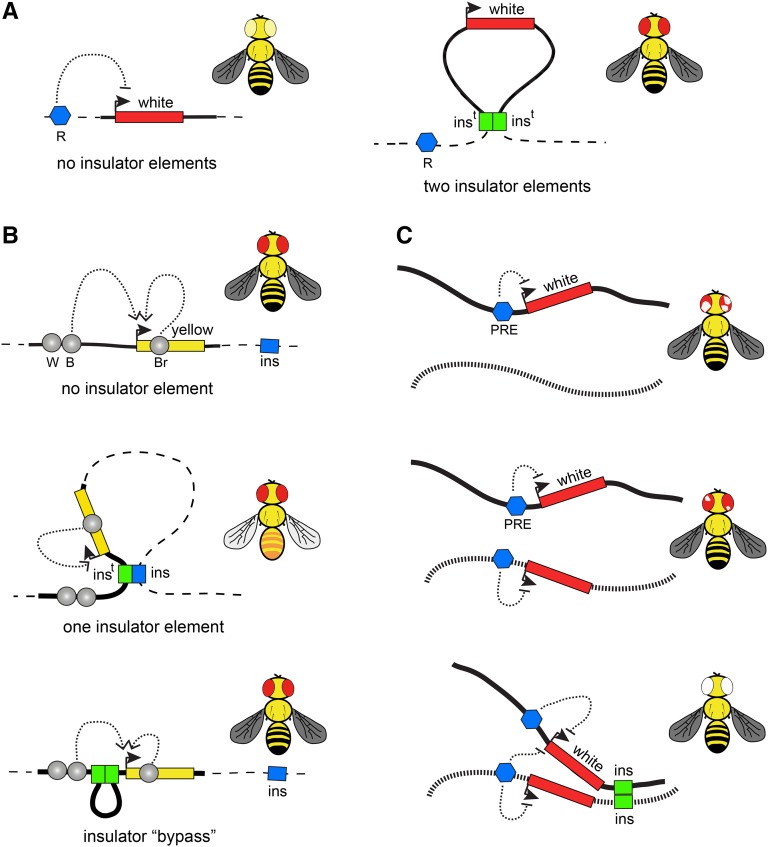
Transgenic insulator assays. (A) Position-effect assay. The *white* gene (red rectangle) confers red pigmentation to fly eyes. When integrated elsewhere in the genome, *white* is frequently repressed by neighboring repressor elements (blue pentagon, R) yielding flies with pale yellow eyes. When a transgene (here and below shown as bold line) carries insulators (green rectangles, ins^t^) at its 5′ and 3′ ends, the *white* expression becomes much more uniform between different insertion sites. (B) Enhancer blocking assay. One of the common enhancer-blocking assays uses the *yellow* reporter gene (yellow rectangle), which controls dark pigmentation of the fly. The expression of *yellow* in wings, body, and bristles is controlled by distinct enhancer elements (gray circles marked with B, W, and Br, respectively). When flies lacking endogenous *yellow* function are transformed with a copy of the WT *yellow* gene, their pigmentation fully restores (black wings, body, and bristles). In contrast, when *yellow*-deficient flies are transformed with a transgene that contains an insulator (green rectangle, ins^t^) interposed between the upstream wing- and body-specific enhancers and the *yellow* promoter, the transgenic insulator interacts with the nearest genomic insulator (blue rectangle, ins) to form a loop that blocks enhancer–promoter communication. Note that the interactions between the promoter and the downstream bristle-specific enhancer are not impaired. This yields transgenic files that have yellow wings and body but black bristles. When two insulators are placed between the upstream enhancers and the promoter, they preferentially interact with each other and stimulate rather than inhibit enhancer–promoter interactions. Corresponding transgenic flies appear as WT. (C) An example of long-distance *trans*-interactions enhanced by insulator elements. In transgenes containing *white* paired with a PRE (blue pentagon), the *white* gene gets stochastically inactivated in embryonic precursor cells, which results in flies with variegated eye pigmentation. When two such transgenes, integrated in different nonhomologous chromosomes (illustrated as black and dashed lines), are combined, the variegation becomes much less pronounced or even disappears. Strikingly, when in addition to PREs the two transgenes also contain insulator elements, the *white* repression is greatly enhanced, often resulting in flies with completely white eyes. This suggests that insulator elements promote long-distance *trans*-interactions and that pairing of PREs reinforces Polycomb repression.

Segmentation of the fly genome into linear and topological chromatin domains raises the question of whether special kinds of elements or structures define the transition from one domain to another. The first attempt to discover such elements nearly 30 years ago, was motivated by the question of what limits the extent of the polytene chromosome region decompacted upon activation of the *Hsp70* genes after heat stress (so-called “heat-shock puff”). Using DNase I accessibility assay, Udvardy *et al.* (1985) have found *specialized chromatin structures* (*scs*) and *scs*′ to flank the *Hsp70* locus and proposed that those define the boundaries of the heat-shock puff. As would be expected from such boundary elements, *scs* and *scs′* turned out to “insulate” the expression of a reporter gene from the influence of the surrounding chromatin (Kellum and Schedl 1991) (Figure 2A). Around the same time, studies of the mutagenic effect of the *gypsy* retrotransposon revealed an insulator element contained in its 5′ end (Holdridge and Dorsett 1991; Geyer and Corces 1992). This element blocks, or insulates, activation of a promoter by a transcriptional enhancer when placed between the two (Figure 2B). Unlike transcriptional repression, insulation leaves the promoter transcriptionally competent, such that it can be activated by other enhancers when they are not separated from the promoter by the insulator element. The two lines of research converged when, similarly to *gypsy* insulator, *scs* and *scs′* were shown to block enhancer–promoter communications (Kellum and Schedl 1992; Kuhn *et al.* 2003) and the *gypsy* insulator was shown to protect a reporter gene from chromosomal position effects (Roseman *et al.* 1993). Another set of paradigmatic insulator elements was discovered while dissecting the regulation of the *Drosophila* bithorax gene cluster. The bithorax complex contains three genes *Ubx*, *abd-A*, and *Abd-B*, which encode transcription factors that specify anterior–posterior identity of the last thoracic and the abdominal segments of the developing fly (Maeda and Karch 2006). The three genes are controlled by an ∼300-kb region, which is divided by insulator elements into nine functionally independent regulatory units (Galloni *et al.* 1993; Karch *et al.* 1994; Mihaly *et al.* 1997; Barges *et al.* 2000; Bender and Hudson 2000; Bender and Lucas 2013; Savitsky *et al.* 2016).

Insulator elements exert their function via associated chromatin insulator proteins and much of what we know about them was discovered in studies of the paradigmatic insulator elements described above. For example, the function of *gypsy* insulator requires the Su(Hw), Mod(mdg4), and Cp190 proteins (Georgiev and Gerasimova 1989; Geyer and Corces 1992; Pai *et al.* 2004) and the BEAF-32 and Dwg (also known as Zw5) proteins are integral components of the *scs′* and *scs* insulators (Zhao *et al.* 1995; Gaszner *et al.* 1999). Overall, the known *Drosophila* insulator proteins can be divided into three groups based on their biochemical and functional properties. The first group contains sequence-specific DNA binding proteins: Su(Hw), CTCF, BEAF-32, Ibf1, Ibf2, Pita, ZIPIC (also known as CG7928), Dwg, and GAF (the product of the Trithorax-like gene) (Geyer and Corces 1992; Zhao *et al.* 1995; Gaszner *et al.* 1999; Moon *et al.* 2005; Cuartero *et al.* 2014; Maksimenko *et al.* 2015; Wolle *et al.* 2015). The second group consists of the Cp190 protein and multiple protein isoforms encoded by the *mod*(*mdg4*) gene (Dorn *et al.* 2001; Pai *et al.* 2004; Van Bortle *et al.* 2012). Both Cp190 and Mod(mdg4) appear to lack sequence-specific DNA binding activity but can mediate homotypic and heterotypic protein–protein interactions via their BTB/POZ (Broad complex, Tramtrack, Bric-a-brac)/(Poxvirus and Zinc finger) domains. The third group contains biochemically diverse proteins: Elba1, Elba2, Elba3, Shep, and Rump, which were proposed to modulate the enhancer-blocking ability of insulator elements at specific stages of development or in a tissue-specific manner (Aoki *et al.* 2012; Matzat *et al.* 2012; King *et al.* 2014). Most of these proteins are specific to diptera, but at least one of them, CTCF, is widely conserved in evolution and, in mammals, acts as the main insulator protein (Herold *et al.* 2012).

Insulator proteins bind chromatin in distinct combinations (Negre *et al.* 2010b; Schwartz *et al.* 2012), often as parts of multisubunit complexes (Pai *et al.* 2004; Cuartero *et al.* 2014; Maksimenko *et al.* 2015). It appears that only sites cobinding certain combinations of insulator proteins can act as robust enhancer blockers (Schwartz *et al.* 2012). This suggests that some insulator proteins may have functions unrelated to chromatin insulation. Indeed, genetic analyses indicate that sites bound by Su(Hw), but not any of the other known insulator proteins, act as transcriptional repressor elements (Schwartz *et al.* 2012; Soshnev *et al.* 2013).

How insulator elements block enhancer–promoter communications is not entirely clear. According to the most popular hypothesis, insulator elements interact with each other and form chromatin loops, which compete with chromatin looping required for enhancer–promoter communication. Indeed there is ample evidence that pairs of insulator elements interact. It was first noted, that while the single *gypsy* insulator placed between the enhancer and promoter of a transgenic reporter gene blocks their communication, the blocking activity is lost when the two *gypsy* insulators are used in place of one (Cai and Shen 2001; Muravyova *et al.* 2001). This “insulator bypass” effect is explained by assuming that a single transgenic *gypsy* insulator interacts with the nearest genomic insulator to form a loop that obstructs the enhancer–promoter communication (Figure 2B). When two *gypsy* insulators are placed between enhancer and promoter, they would preferentially interact with each other and form a loop, which would shorten the distance between enhancer and promoter, stimulating rather than inhibiting their interaction (Cai and Shen 2001; Muravyova *et al.* 2001). Pairwise interactions are not limited to *gypsy* insulators. In fact, the majority of tested insulator elements appear to interact when paired up. However such interactions vary in strength and sometimes are detected only by more sensitive transgenic assays based on stimulation of short-range enhancers (Kuhn *et al.* 2003; Gruzdeva *et al.* 2005; Kyrchanova *et al.* 2007, 2008a,b; Fujioka *et al.* 2016). The interactions are often directional and can happen between two distinct elements (Kyrchanova *et al.* 2008a; Fujioka *et al.* 2016). For specific subclasses of insulator elements, the looping interactions have been demonstrated at the molecular level (Blanton *et al.* 2003; Comet *et al.* 2011). However, more work is needed to understand the factors that define combinations of distinct insulator elements that can interact.

Interactions between insulator elements are not limited to pairs contained within one transgenic construct (Figure 2C). Several elegant studies indicate that interaction between insulator elements can mediate the transvection between loci located hundreds of thousands of base pairs apart or even on different chromosomes (Kravchenko *et al.* 2005; Fujioka *et al.* 2016). Likewise, insulators were shown to mediate long-distance interactions and enhance repression of reporter genes by PREs (Li *et al.* 2011, 2013). In this view, the same kinds of interactions that lead to blocking enhancer–promoter communications also bring different genomic elements together and juxtapose regulatory elements with target promoters. The 3D contacts facilitated by insulator elements are likely transient, implying that the role of insulators is to increase the probability of certain chromatin conformations rather than generating a rigid loop structure.

The molecular mechanics of insulator interactions is a subject of active investigation. According to the mainstream model, the sequence-specific DNA binding insulator proteins of the first group serve as adaptors to recruit proteins of the second group, which act as a “glue” to hold different insulator elements together. Consistently, both Cp190 and Mod(mdg4), the candidate glue proteins, share genomic binding sites with many DNA binding proteins of the first group (Negre *et al.* 2010b; Schwartz *et al.* 2012; Van Bortle *et al.* 2012). Both proteins also form distinct nuclear foci, which may correspond to clusters of multiple insulator elements held together by Cp190 or Mod(mdg4) (Gerasimova *et al.* 2000, 2007). This view, however, is contested with the alternative hypothesis that the Cp190 and Mod(mdg4) foci represent aggregates of proteins not bound to chromatin (Golovnin *et al.* 2008, 2012). The distinction between the glue and the sequence-specific recruiter proteins may not be as clear cut. Recent studies suggest that the DNA binding proteins CTCF, Dwg, Pita, and ZIPIC can homodimerize. This may also contribute to interactions between insulator elements (Bonchuk *et al.* 2015; Zolotarev *et al.* 2016). The Cp190 and Mod(mdg4) proteins, themselves, are biochemically and functionally different. Although both proteins have BTB/POZ domains, these domains differ in their interaction preferences. In the yeast two-hybrid and *in vitro* assays, the BTB/POZ domain of Mod(mdg4) forms homo- and heterotypic multimers with the BTB/POZ domains of several other members of the tramtrack group (Golovnin *et al.* 2007; Bonchuk *et al.* 2011), while the BTB/POZ domains of Cp190 only form homodimers (Bonchuk *et al.* 2011; Vogelmann *et al.* 2014). Moreover, the isolated BTB/POZ domains of Cp190 and Mod(mdg4) do not interact with each other. Consistent with their diverse biochemical properties, mutations in the *Cp190* and *mod*(*mdg4*) genes have distinct phenotypes (Savitsky *et al.* 2016). To summarize, it appears that the biochemical combination of insulator proteins bound to an insulator element defines the range of potential insulator elements it can interact with and, possibly, the directionality of these interactions. More work is needed to test this hypothesis and elucidate the combinatorial rules.

How could the elements that facilitate transient looping contacts delimit the boundaries of combinatorial patterns of chromatin modifications (chromatin states)? One scenario that is easy to envision is when histone modifications are produced via looping interactions of a protein complex anchored at a fixed chromatin site. For example, Polycomb complexes anchored at PREs (see below for details) loop out and trimethylate H3K27 at extended distances from their principal binding sites (Kahn *et al.* 2006; Schwartz *et al.* 2006). The “spreading” of H3K27me3 from PREs can be blocked by chromatin insulator elements due to the reduction of transient looping contacts between the PRE-anchored complexes and surrounding chromatin (Kahn *et al.* 2006; Comet *et al.* 2011). Similar to the enhancer blocking case, a pair of interacting insulator elements can be bypassed, leaving the stretch of chromatin between the insulators free of H3K27me3 with the high level of H3K27 methylation in chromatin further away from the insulator pair (Comet *et al.* 2011). Genomic studies indicate that insulator elements do restrict the spreading of H3K27me3 domains around Polycomb target genes (Bartkuhn *et al.* 2009; Schwartz *et al.* 2012). However, their contribution is most critical to prevent the methylation of the neighboring genes that are transcriptionally inactive (Schwartz *et al.* 2012) because chromatin remodeling linked to transcriptional activity can, by itself, inhibit H3K27 methylation. For example, histone H3 molecules methylated at lysine 4 or lysine 36 are poor substrates for histone methyltransferase activity of the PRC2 complexes (Schmitges *et al.* 2011; Yuan *et al.* 2011; Voigt *et al.* 2012). When histone modifications are produced in the immediate vicinity or by processive movement of an enzyme along the chromatin fiber, for instance by an enzyme linked to transcribing RNA polymerase, insulator elements would have little effect on their spreading. It is, therefore, not surprising that the boundaries of domains with distinct chromatin states (combinations of histone modifications) show only limited overlap with the insulator protein binding sites (Kharchenko *et al.* 2011; Schwartz *et al.* 2012).

Thanks to the remarkable property to form *trans*-interactions, insulator elements are naturally expected to play a role in partitioning the *Drosophila* genome into TADs. Indeed, the partitioning of the bithorax complex cluster of homeotic genes into distinct TADs by the *Fub* insulator element represents one such beautiful example (Bender and Lucas 2013; Savitsky *et al.* 2016). However, what fraction of TADs is defined by insulator elements remains an open question. Ironically, although the quest to define specific chromatin boundary elements started with the suggestion that *scs* and *scs′* insulators delimit the extent of the decondensed chromatin domain of the *Hsp70* locus, careful microscopy measurements showed that *scs* and *scs′* reside well within and not at the borders of the *Hsp70* puff (Kuhn *et al.* 2004). Comparison of TADs defined by the Hi-C approach to genomic distributions of insulator proteins indicates that Cp190 and BEAF-32 frequently colocalize with TAD borders (Hou *et al.* 2012; Sexton *et al.* 2012; Ulianov *et al.* 2016). This can be taken to indicate that insulator proteins define the TAD limits. However, the interpretation is likely more complex. A large fraction of Cp190 and BEAF-32 binding sites are in the vicinity of TSSs of active genes (Bushey *et al.* 2009; Schwartz *et al.* 2012) and whether these Cp190 and/or BEAF-32 binding sites correspond to insulator elements is unknown. It is important to keep in mind that TAD borders are defined as points at which the frequencies of interactions between adjacent chromatin stretches change. Since transcriptionally active and inactive chromatin display distinct folding regimes (Sexton *et al.* 2012; Boettiger *et al.* 2016), the transition between the two kinds of chromatin is likely to appear as TAD boundary. Consistently, combinations of histone modifications typical of active genes can predict the positions of many *Drosophila* TAD borders (Ulianov *et al.* 2016). It is therefore possible that overrepresentation of Cp190 and BEAF-32 at TAD borders simply reflects their bias toward active TSSs.

How many of the TAD borders depend on insulator elements is not entirely clear. In principle, Hi-C profiles of TADs defined by looping interactions of two insulator elements should carry distinct signatures of enhanced contact frequencies between the insulator elements. Those would appear as brighter “dots” at the top of the corresponding TAD “pyramids” (Figure 1). Indeed, such high-contact foci corresponding to enhanced contacts between some of the CTCF binding sites are clearly visible in the high-resolution contact map of the human genome (Rao *et al.* 2014). For some reason, the foci of enhanced contacts are not as readily detectable in the *Drosophila* Hi-C maps (Hou *et al.* 2012; Sexton *et al.* 2012; Eagen *et al.* 2015; Ulianov *et al.* 2016).

Future experiments that look at changes in genome topology in cells lacking key insulator proteins will tell us how many of the TAD borders depend on insulator elements. Until then, it is safe to conclude that a fraction of TAD borders is formed by insulator elements and these borders are essential for faithful regulation of genes with complex regulatory regions. The role of the insulator-based borders is likely most critical in cells where they separate TADs that cover genes with similar transcriptional states. For example, an insulator element placed between a TAD encompassing a Polycomb-repressed gene and a TAD with a transcriptionally inactive gene is needed to prevent the latter from being permanently repressed.

## Polycomb Complexes: Linking Epigenetic Regulation with 3D Chromatin Organization

PcG proteins are a group of chromatin regulatory components that are able to modulate or silence the expression of euchromatic genes. First discovered in 1947 by Pamela Lewis (Lewis 1947) and originally believed to be one of the homeotic (Hox) genes that specify the anterior–posterior body plan, the *Polycomb* (*Pc*) gene was then described as a separate locus that represses Hox genes outside their appropriate expression domains (Lewis 1978). Other genes were soon shown to have similar functions as *Pc* (Struhl and Brower 1982; Jürgens 1985). Furthermore, a seminal discovery was that, in contrast to early patterning transcription factors whose dysregulation disrupts the initiation of the correct segmental expression of Hox genes, mutations in Polycomb Group (PcG) genes initially show little or no phenotypes but, later, induce Hox gene derepression (Struhl and Akam 1985). Screening for suppressors of PcG function led to the discovery of a set of genes that counteract PcG-mediated silencing, named trithorax-group (trxG) genes after the first member of the group (Ingham 1983; Kennison and Tamkun 1988; Tamkun *et al.* 1992).

Analysis of polytene chromosomes showed that PcG proteins bind to Hox loci and to a variety of other loci in the genome (Zink and Paro 1989; Rastelli *et al.* 1993), indicating that their functions may be more widespread than Hox gene regulation. Furthermore, cloning of *Pc* showed that its protein possesses a short region, defined as chromo domain, very similar to that of the HP1 protein (Paro 1990; Paro and Hogness 1991). The genetically defined function in maintenance of gene silencing and the conservation of the chromo domain with HP1, which was involved in heterochromatin maintenance, suggested that PcG genes may somehow set up a memory of cell transcription states (Paro 1990). This memory function was later demonstrated using transgenes containing PcG binding sites (Cavalli and Paro 1998, 1999; Maurange and Paro 2002; Poux *et al.* 2002; Bejarano and Milan 2009).

**Figure 3:**
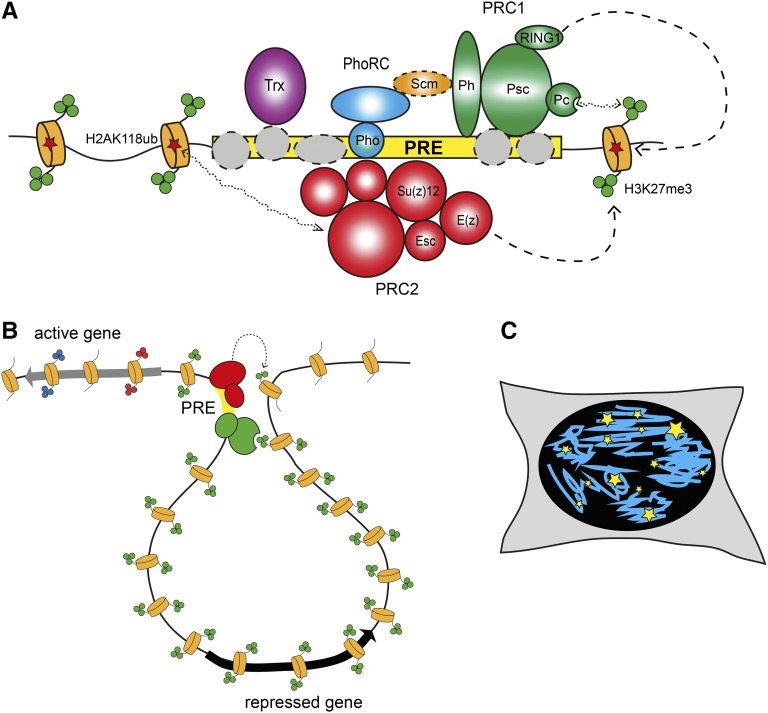
Polycomb complexes and their role in chromatin architecture. (A) Polycomb complexes are targeted to genes by PREs (yellow rectangle). PREs represent collections of recognition sequences for DNA binding adaptor proteins (gray circles). With the exception of Pho, which is part of the separate PhoRC complex, DNA binding proteins interact with the core PcG complexes weakly and are not recovered in biochemical purification. Nevertheless, individual weak interactions of DNA binding proteins combine and provide robust recruitment of PRC1 (green circles) and PRC2 (red circles). Scm (orange oval), the substoichiometric component PRC1, serves as a link between PhoRC and PRC1, further stabilizing the binding of both complexes (Kahn *et al.* 2014; Frey *et al.* 2016). Chromodomain of the Pc subunit of PRC1 specifically interacts with trimethylated H3K27 produced by PRC2 (green circles on the N-terminal tails of nucleosomes depicted as orange cylinders). In turn, PRC2 can interact with monoubiquitylated H2AK118 produced by PRC1 (red stars). Although insufficient for recruitment, these interactions can further stabilize the binding of PRC1 and PRC2 at some PREs. The Trx protein is recruited to PREs along with PcG complexes. (B) PRC1 (red circles) and PRC2 (green circles) complexes anchored at PREs (yellow rectangle) loop out and contact surrounding chromatin. Recognition of H3K27me3 by Pc stabilizes these transient interactions, allowing efficient methylation of nucleosomes in the vicinity of the contact. This way H3K27me3 can spread away from a PRE for tens of thousands of base pairs until it encounters an insulator element or an active gene. (C) Immunolocalization of Polycomb components in *Drosophila* or mammalian cell nuclei detects discrete foci of different sizes (PcG bodies, yellow stars). The number of PcG bodies is smaller compared to the number of Polycomb target genes detected by genome-wide mapping. Immuno-FISH experiments suggest that some of the PcG bodies represent clusters of Polycomb-regulated genes.

Indeed, PcG proteins were shown to bind to specific regulatory elements of about 1000 bp. These polycomb response elements (PREs) could recruit PcG proteins also when they were inserted in transgenic constructs, where they could drive repression of reporter genes (Busturia and Bienz 1993; Fauvarque and Dura 1993; Simon *et al.* 1993; Chan *et al.* 1994; Zink and Paro 1995). The analysis of PRE-containing transgenes showed that PcG-mediated silencing has properties similar to those of heterochromatin, such as a variegated silencing of the mini-*white* reporter gene. However, two notable differences are that higher temperatures enhance PcG-dependent silencing, whereas they reduce heterochromatin silencing, and that homologous pairing of PRE-containing transgenes induced increased silencing efficiency at a subset of the transgene insertion loci (Pirrotta and Rastelli 1994). Since the identification of genomic elements necessary and sufficient for PcG targeting, much effort has been dedicated to reveal how the targeting happens at the molecular level. The results of this large body of work were summarized in an excellent recent review (Kassis and Brown 2013). Briefly, PREs seem to represent collections of recognition sequences for multiple DNA binding adaptor proteins (Figure 3A). With an exception of Pleiohomeotic (Pho) or closely related Pleiohomeotic-like (Phol) proteins (Brown *et al.* 1998, 2003), which form a distinct PhoRC complex (Klymenko *et al.* 2006), the other DNA binding proteins interact with the core PcG complexes too weakly to be recovered as stoichiometric components of the complexes. It was proposed that individual weak interactions of the DNA binding proteins combine to provide robust recruitment of Polycomb repressive complexes 1 and 2 (PRC1 and PRC2, see below).

At the beginning of the 1990s, laboratories studying mammalian development became interested in this intriguing set of proteins and they identified mammalian homologs of fly PcG proteins (Pearce *et al.* 1992; Ishida *et al.* 1993; Muller *et al.* 1995). Strikingly, trxG genes were also shown to be conserved (Djabali *et al.* 1992). Furthermore, homology of the two fly members of the PcG *Psc* and *Su*(*z*)*2* to a murine protooncogene named Bmi-1 strongly suggested that, in addition to its requirement during development, the appropriate regulation of PcG function is also required to prevent the emergence of cancer. Since then, countless reports have corroborated this hypothesis (Koppens and van Lohuizen 2016), extended the link with cancer to trxG proteins (Schuettengruber *et al.* 2011) and, finally, the appropriate regulation of fly PcG components has also been demonstrated to prevent oncogenesis (Classen *et al.* 2009; Martinez *et al.* 2009; Sievers *et al.* 2014). These findings have raised the interest of the scientific community for PcG and trxG proteins considerably.

Similar phenotypes observed in PcG mutants and the colocalization of PcG proteins in polytene chromosomes strongly suggested that PcG proteins act in concert, possibly via formation of biochemical complexes, to recognize repressed transcriptional states and to propagate them through cell division. Furthermore, their increased silencing activity in the presence of homologous PREs suggested that long-range interactions in the 3D nuclear space may reinforce silencing. Co-immunoprecipitation studies provided first evidence for molecular interactions among *Drosophila* and mammalian PcG components (Franke *et al.* 1992; Alkema *et al.* 1997). This was followed by the isolation of the *Drosophila* PcG complex dubbed Polycomb repressive complex 1 (PRC1). This large complex contained Pc, Polyhomeotic (Ph), Posterior Sex Combs (Psc), and RING1 (the product of the Sex Combs Extra gene) as stoichiometric components, as well as Sex Combs on Midlegs (Scm) in substoichiometric amounts (Shao *et al.* 1999). PRC1 was shown to have chromatin condensation activity (Francis *et al.* 2004), as well as ubiquitylation activity toward lysine 118 (119 in mammals) of histone H2A (de Napoles *et al.* 2004; Wang *et al.* 2004; Cao *et al.* 2005). Soon after the discovery of PRC1, the PRC2 was isolated both in flies and mammals as a complex containing the Enhancer of zeste [E(z)], Suppressor of zeste 12 [Su(z)12] and Extra Sex Combs (Esc) proteins. PRC2 is able to mono-, di-, and trimethylate lysine 27 of histone (H3K27) with E(z) acting as catalytic subunit (Cao *et al.* 2002; Czermin *et al.* 2002; Kuzmichev *et al.* 2002; Muller *et al.* 2002). This histone modification was shown to be specifically recognized by the Chromo domain of Polycomb, establishing a first link between PRC2 and PRC1 complexes (Fischle *et al.* 2003). More recently, the reverse possibility that PRC1-dependent H2AK118Ub is recognized by PRC2 has also been suggested (Blackledge *et al.* 2015). This double link might generate a self-enforcing loop whereby recognition of H2AK118Ub by PRC2 would stabilize its binding while the recognition of H3K27me3 by PRC1 may help to spread H3K27me3 around PREs (Figure 3B). Although this is an appealing idea, which could explain the robustness of PcG-mediated repression, the link between PRC1-mediated H2A ubiquitylation and PRC2 recruitment has been called into question and further investigations are required to clarify the issue (Lee *et al.* 2015; Pengelly *et al.* 2015). Finally, it is important to realize that some Polycomb genes have one or more paralogs. In flies, this is true for one PRC2 member, *esc*, which has a paralog called *escl* (Wang *et al.* 2006; Ohno *et al.* 2008), but also for the *ph* and *Psc* loci (Grimaud *et al.* 2006b; Schuettengruber *et al.* 2007). Mammalian PcG components are even more redundant, with several paralogs for each of the subunits (Piunti and Shilatifard 2016). Detailed work in mammals has identified a whole series of PRC1-related complexes. Canonical PRC1 variants (cPRC1) are those that contain homologs to each of the original PRC1 components isolated in flies, *i.e.*, PC, PH, PSC, and SCE, whereas in noncanonical PRC1 variants, PC and PH homologs are absent and the ncPRC1-specific RYBP proteins are present instead. Furthermore, specific types of PSC paralogs characterize specific ncPRC1 variants and other proteins are present in some of them (Gao *et al.* 2012). While most of the members of ncPRC1 are conserved in flies, whether they form similar complexes and their function is conserved is still unclear. More studies are required to address these questions.

In parallel to the biochemical characterization of PcG complexes, a large body of work has been dedicated to describe and understand their genome-wide distribution. Initial work in flies paralleled human and mouse studies (Boyer *et al.* 2006; Lee *et al.* 2006; Negre *et al.* 2006; Schwartz *et al.* 2006; Tolhuis *et al.* 2006). These studies showed that PRC1 and PRC2 components colocalize at a set of developmental regulatory target genes, many of them coding for developmental transcription factors, including a large set of homeodomain-containing sequence-specific DNA binding proteins. Strikingly, many of these target genes are linked and regulate multiple steps of transcription regulatory cascades that regulate developmental patterning or cell differentiation (Schuettengruber *et al.* 2007; Schwartz and Pirrotta 2007). In the early mammalian studies, a subset of PcG targets in ES cells was shown to be bivalent, *i.e.*, simultaneously decorated by the activating mark H3K4me3 (Boyer *et al.* 2006; Lee *et al.* 2006). Of note, H3K4me3 deposition depends on TrxG complexes called COMPASS, which are conserved from flies to human (Piunti and Shilatifard 2016). Although in flies little evidence exists for the existence of bivalent genes, subsequent work has shown that trxG components are frequently cobound at PcG target genes, illustrating the collaboration/competition between PcG and TrxG components in the fly genome (Schuettengruber *et al.* 2009; Schwartz *et al.* 2010; Enderle *et al.* 2011). Although many of the targets are consistently found in all cell types, other targets are dynamically bound and regulated, such as the Notch gene, which is bound and repressed by the PH component of PRC1 in larval development but not in embryos (Martinez *et al.* 2009). Indeed a recent study has shown that PcG binding is dynamic during development and that two types of PcG target genes exist: canonical targets carrying PRC1 and PRC2 binding in the presence of the H3K27me3 mark, and a novel category, defined as neo-PRC1 genes, which include the Notch gene and are bound by PRC1 and PRC2 in the absence of its H3K27me3 mark (Loubiere *et al.* 2016).

One intriguing observation that came from the comparison of genome-wide location with microscopy studies, done either by immunofluorescence or by analysis of GFP-fusion proteins of the PcG, is that PcG components stain as foci in the nucleus (Buchenau *et al.* 1998; Grimaud *et al.* 2006a; Terranova *et al.* 2008; Cheutin and Cavalli 2012) (Figure 3C). Although any comparison between microscopic foci and genome-wide binding sites is difficult, the detection of a rather limited number of nuclear foci suggested the possibility that long-range contacts might exist between PcG-bound elements. This idea, also supported by the phenomenon of pairing sensitive silencing in PRE-containing transgenes, was directly tested by DNA FISH analysis of the 3D nuclear location of PRE-containing transgenes. Initially, transgenes containing one regulatory region called *Fab-7*, which contains a PRE flanking a chromatin insulator, were shown to be able to frequently contact the endogenous *Fab-7* element even when inserted in a different chromosome (Bantignies *et al.* 2003). The combination of FISH with immunostaining showed that this colocalization occurred at PcG foci and was dependent on PcG proteins, as well as on RNA interference components, although the molecular mechanism linking these proteins to PcG members could not be elucidated (Grimaud *et al.* 2006a). Later, other transgenes were shown to be capable of inducing contacts and the insulator elements flanking PREs were demonstrated to play a pivotal role in targeting 3D interactions (Li *et al.* 2011). The action of insulators seems to be neutral with regard to the regulatory outcome, *i.e.*, insulators can drive associations in the 3D space of the cell nucleus of their target elements. If these elements are in a repressed state, their association is accompanied and possibly stabilized by PcG components, whereas if they are active, TrxG proteins assist their association to transcriptionally active regions of the nucleus (Li *et al.* 2013).

These data suggested that a cooperation between PcG proteins, TrxG proteins, and insulators may lead to higher-order organization of a large set of genomic loci. This idea is at least partly supported by several lines of evidence. First, the analysis of nuclear positioning of Hox genes has shown that the bithorax and the antennapedia complexes colocalize in nuclei in which Hox genes are corepressed (Bantignies *et al.* 2011). Microscopic colocalization signifies chromatin contacts, as shown by 4C studies both in embryos and in larval brains (Bantignies *et al.* 2011; Tolhuis *et al.* 2011). Furthermore, 4C and Hi-C studies identified a large network of contacts involving PcG target loci (Bantignies *et al.* 2011; Tolhuis *et al.* 2011; Sexton *et al.* 2012). Importantly, reducing the frequency of Hox gene contacts by mutating regulatory elements in the bithorax complex, induced the derepression of Hox genes in the antennapedia complex, which is located 10 Mb away (Bantignies *et al.* 2011). This result suggests that clustering of PcG target genes may stabilize silencing. Furthermore, recent evidence also suggests that 3D architecture may also stabilize recruitment of Polycomb proteins (Schuettengruber *et al.* 2014; Entrevan *et al.* 2016). It is noteworthy that contacts between genomic regions bound by PcG components are conserved in mammalian cells (Schoenfelder *et al.* 2015; Vieux-Rochas *et al.* 2015), suggesting that the 3D aspect of PcG biology is also a conserved feature. Obviously, it is of great importance to understand the mechanisms regulating clustering of PcG sites in space. Initial work suggests that the PH subunit of PRC1 may be important for this process, both in flies and in mammals (Isono *et al.* 2013; Wani *et al.* 2016). Clearly, much more work has to be done to understand the mechanisms of PcG-mediated gene contacts, those that govern delimitation of PcG chromatin spreading in the flanking genome, and the interplay between PcG, TrxG components, and other chromosomal proteins to regulate nuclear organization of their target genes.

## Conclusions/Perspectives

For decades *Drosophila* has been a pioneering model system to identify and describe the connection between 3D organization of the genome and its function. Some important aspects of this organization, for instance, the compartmentalization of the genome parts to specific subnuclear locations, such as the nuclear lamina or the nucleolus, were not discussed in this review due space limitations. Nevertheless, the examples provided here clearly show that the genome cannot be reduced to a string of DNA nucleotides and that all levels of higher-order genome organization, from the nucleosome to chromatin fibers, chromosomal domains, chromosome territories, and chromosome localization within nuclear space, must be considered. A great amount of work is still required to dissect the links between each of these organizational levels and various aspects of genome function. Part of this work will likely involve the analysis of chromosome architecture of specific cell types or developmental stages, similar to the recent analysis of the spatial regulation of the bithorax complex along the anteroposterior body plan during embryogenesis (Bowman *et al.* 2014). To this aim, the development of low- or single-cell techniques is needed, since most of these studies do not allow isolation of large amount of homogeneous cells.

**Figure 4:**
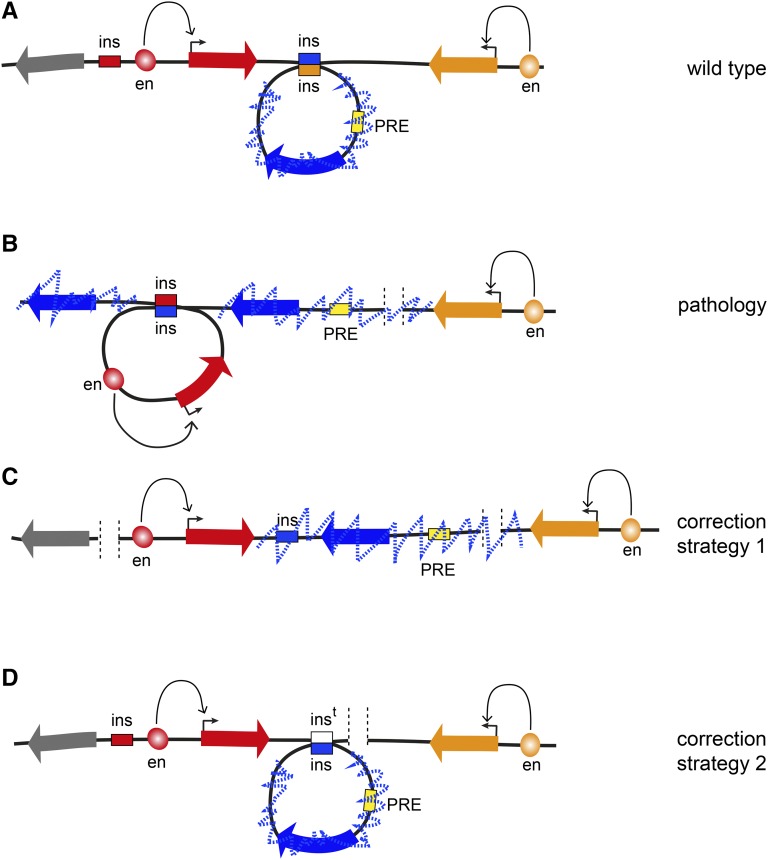
Prospects of 3D genome engineering. (A) In a fictional WT locus, the transcription of the “red” and the “orange” genes is driven by specific enhancers. The leftmost “gray” gene is inactive but is not epigenetically repressed and could be activated later during development. The “blue” gene is surrounded by insulators (blue and orange boxes), which interact, forming a looped TAD. A PRE located within the TAD (yellow box) represses the blue gene and leads to extensive trimethylation of H3K27 (dashed blue zig-zag line) that is contained within the TAD. (B) When the rightmost orange insulator is deleted by mutation, the central blue insulator loses its preferred interaction partner and instead interacts with the leftmost red insulator, forming the TAD containing the active red gene. The PRE is no longer topologically constrained. This leads to extended H3K27 methylation to the right of the PRE that stops at the transcriptionally active orange gene. It also leads to the insulator bypass, H3K27 methylation, and epigenetic repression of the leftmost gene. This gene can no longer be induced, which causes pathology later in development. (C) Using genome editing tools, for example CRISPR/Cas9, it may be possible to delete the red insulator. This will prevent formation of the TAD containing the red gene and its transcriptional activity will stop the spreading of H3K27me3 and prevent the leftmost gray gene from being permanently repressed. (D) Ultimately, it might be possible to use more sophisticated homology-directed replacement techniques to reintroduce a new copy of the deleted insulator (indicated as white box) and fully restore the WT topology of the locus.

As studies that connect disruptions of TADs to pathological rewiring of enhancer–promoter interactions and heritable malformations in human patients started to emerge (Lupianez *et al.* 2015), there is mounting pressure to understand the basic rules that govern genome folding. Knowing these rules will advance our ability to interpret consequences of deletions, duplications, inversions, and translocations that are found within normal human population. Many of these structural variations are linked to predisposition to disease. In cases when a variation changes gene dosage the link is easier to explain. In contrast, the consequences of inversions or rearrangements that affect noncoding DNA are much more difficult to predict unless we know how they may impact genomic interactions. Once the principles that govern genome architecture are charted, we will be in position to correct some of the 3D aberrations using advances from the burgeoning field of precision genome editing (Figure 4). We have no doubt that *Drosophila* will continue to be instrumental in our quest to achieve this goal.
